# Association of oral health awareness and practice of proper oral hygiene measures among Saudi population: a systematic review

**DOI:** 10.1186/s12903-023-03522-w

**Published:** 2023-10-24

**Authors:** Reham AlJasser, Aljoharah Alsinaidi, Noor Bawazir, Lama AlSaleh, Aseel AlOmair, Haifa AlMthen

**Affiliations:** 1 Department of Periodontic and Community, College of Dentistry, King Saud University, PO Box 60169, Riyadh, 11545 Saudi Arabia; 2 Collage of Dentistry, King Saud University, Riyadh, Saudi Arabia

**Keywords:** Oral health, Oral hygiene, Dental care, Saudi Arabia, Gingivitis, Periodontitis

## Abstract

**Background:**

Several studies have proven that increasing oral hygiene knowledge correlates with good oral health status compared to those who lack this knowledge. Therefore, the aims of the study to evaluate the overall oral health awareness among the Saudi population based on knowledge and practice of proper oral hygiene measures.

**Methods:**

A systematic review was performed according to the Preferred Reporting Items for Systematic Reviews guidelines. Cross-sectional, cohort, and case-control studies were included in the study and framed into a PICO question. Initially, a search was conducted on PubMed/Medline, Google Scholar, and Cochrane databases. Four independent reviewers screened the identified titles, abstracts, and full texts. Cohen’s Kappa score was used to evaluate the level of agreement between the reviewers.

**Results:**

Forty cross-sectional studies and one prospective cohort study were included. Several studies showed that most students across all departments of universities knew the protective effects of fluoride on teeth. Two studies assessing the attitude and practice of oral hygiene found that most students knew that poor oral health leads to gum disease, and 59.1% were aware of maintaining oral hygiene using a toothbrush and paste. Most participants knew the importance of oral and dental care before pregnancy and how to reduce dental problems during pregnancy. Pregnant women clean their teeth daily and consider brushing and using toothpaste essential for pregnant women. Studies on oral hygiene practices of patients with diabetes reported that flossing habits were rated less important and most of their respondents never flossed their teeth.

**Conclusion:**

Strong correlation between oral health knowledge and practices was observed, with the higher the knowledge level, the better the practice. Therefore, new technologies and strategies must be tested for an effective oral health system.

**Trial Registration:**

A protocol was specified and registered with the International Prospective Register of Systematic Reviews (PROSPERO) on August 2020 (registration number CRD 42,020,200,373).

## Background

Maintaining good oral health is crucial and can affect general health and well-being; it can be defined as the lack of oral and facial pain, malignancies, dental infections, and diseases or disorders which negatively affect an individual’s oral l functionality and sociality [1]. Oral hygiene is a crucial element in gaining better oral health. Several studies have proven that increasing oral hygiene knowledge correlates with good oral health status compared to those who lack this knowledge [2].

In India, a systematic review was conducted regarding oral health awareness among workers with angina [3]. Most of the participants in these studies were undergraduates. The authors reported that more than 90% of participants understood the correlation between oral health and physical well-being [3]. However, other studies have reported that less than 10% of participants lack knowledge of the association of smoking with cancer which represents the unawareness of the population [3]. For that reason, it is necessary to carry out regular oral health programs. They also reported that less than 10% of participants are unaware of the anti-cariogenic effect of fluoride [3].

Several Nigerian studies were conducted among parents, schoolteachers, and teenagers to assess their oral health awareness [4]. Authors reported that between 52% and 80% of participants had never visited a dentist [4]. Another study found that 37.8% of participants suffered from oral pain; however, only 12.4% had been to a dentist, while the rest ignored the pain and refused to seek dental treatment due to fear of losing teeth with proposed extractions [4].

In contrast, another study assessed oral health knowledge among pregnant women in Poland, which demonstrated that 40% of participants lacked basic dental knowledge during pregnancy and early childhood [5]. Moreover, they revealed that more than 70% of participants had developed gingivitis or periodontitis [5].

In Saudi Arabia, several studies have discussed this issue among multiple populations. One of these studies was done in the city of Makkah, which proved the positive impact of higher education on the frequency of toothbrushing [6]. They also showed that females brush their teeth more than males; however, males use a miswak which is a teeth-cleaning twig made from the *salvadora persica* tree more than females [6].

They also have revealed that the first exposure to dental care for more than 88% of Saudi children started after seven years old, which explains the high prevalence of dental caries among children and adolescents [6].

Furthermore, a third study was conducted in the Asser region among parents regarding infants’ dental awareness, which showed that 72.62% of parents took care of the oral health of their babies [7]. Approximately 67% claimed that both primary and permanent teeth are essential, and the rest believed there is a difference between both dentitions. Approximately 83% of parents stated that good oral health could enhance physical well-being [7].

Another study assessed the oral health status of children aged 6–13 in south Jeddah [1]. The study showed that approximately 50% had never visited a dentist, and 42% had only visited it for emergencies [1]. Approximately only 7% periodically visited the dentist [1]. They also found that females tend to seek dental treatments more often than males [1]. Moreover, a higher socioeconomic status correlates significantly with an increase in oral health knowledge [1].

Another study conducted among school children in Abha assessed oral health knowledge and practice [8]. It showed that 82% of participants agreed upon the association between oral health and general health [8]. However, most participants (69.6%) do not visit the dentist unless there is an emergency [8].

Overall, several studies have been conducted in multiple regions in the Kingdom of Saudi Arabia to measure awareness of oral health status among their residents. Therefore, the present systematic review aims to evaluate the overall oral health awareness among the Saudi population in all regions based on knowledge and practice of proper oral hygiene measures.

## Methods

### Study design

A systematic review aimed to assess oral health awareness among different Saudi Arabian populations and how it affects oral hygiene; it was performed according to the guidelines set out by the Preferred Reporting Items for Systematic Reviews (PRISMA) [9].

#### Eligibility criteria for study inclusion

Cross-sectional, cohort, and case-control studies were eligible for inclusion. The PICO elements were identified as follows:

- Populations: Saudi Population.
- Intervention: oral health awareness OR oral hygiene knowledge.
- Comparison: None.
- Outcome: tool and index used, e.g., plaque index, bleeding index, and survey scorings.
- And accordingly, the PICO focused question was identified as follows;
  What is the level of oral hygiene awareness when measured from clinical outcomes based on knowledge and practice of proper oral hygiene measures among Saudi population?

### Search strategy

An initial search was conducted of PubMed/Medline, Google Scholar, and Cochrane databases. The search included all reported data until July 2022. Details regarding the search terms are as follows: ((Saudi population or Saudi participants or Saudi patients) AND (oral health or oral hygiene or oral awareness)) AND (plaque index or bleeding index or survey scoring).

There were no language restrictions in searching articles using keywords and MeSH terms. Other relevant terms and Boolean operators (OR, AND) were used to combine searches, and articles were screened without language restrictions. Further hand-searching was done.

### Assessment of validity

**Fig. 1:**
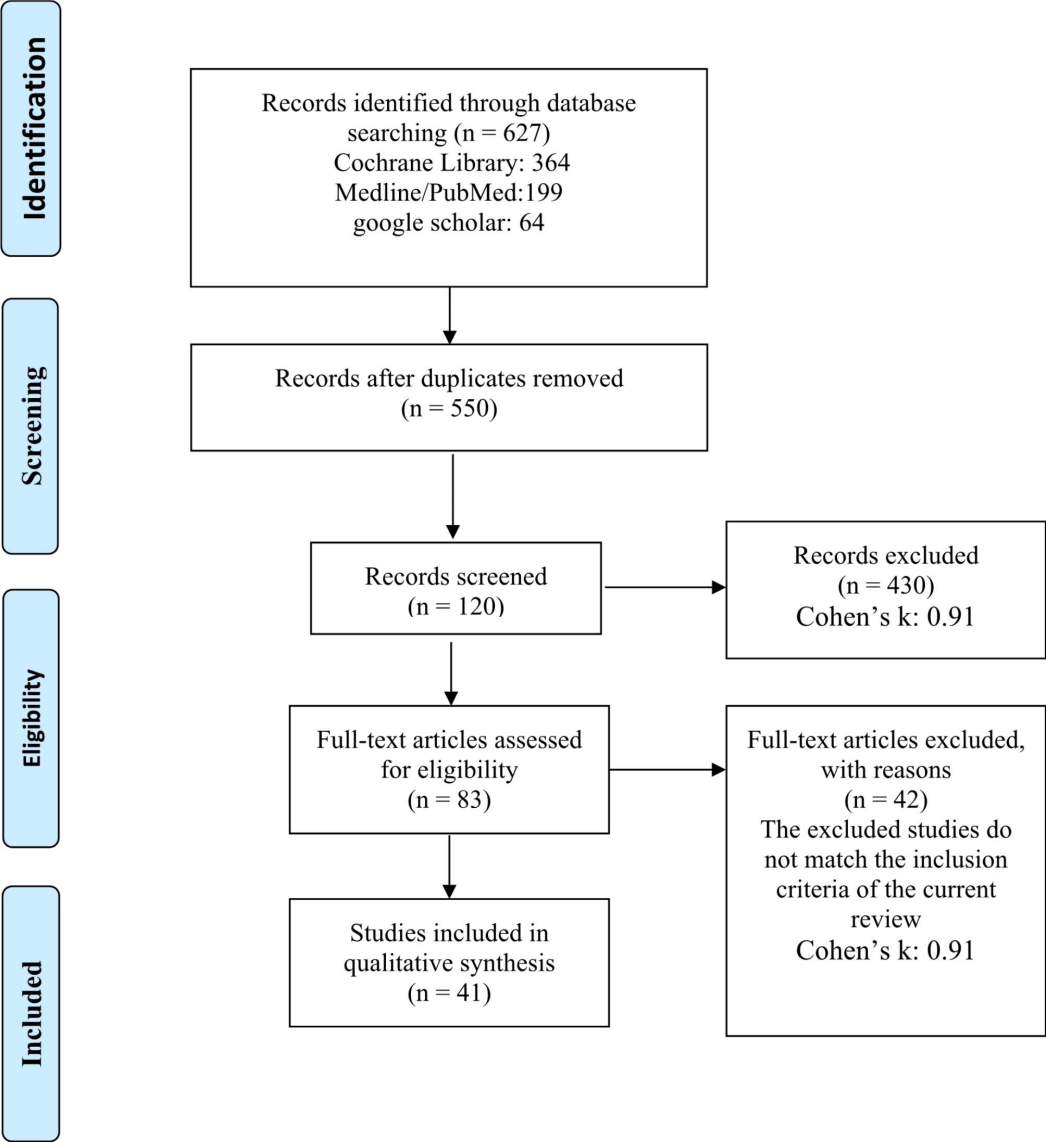
Flow diagram of study selection

Four independent reviewers, NB, LS, AO, and HM, screened the identified titles, abstracts, and full texts. Discussions were held to reach a general agreement on the studies included. During the selection process, Cohen’s Kappa score was used to evaluate the level of agreement between the reviewers. The included data went through data extraction and validity assessment. The reasons for excluding studies were recorded (Fig. 1).

### Data extraction

A pre-designed form was developed to extract the following data: Author name(s); publication year and place; source of funding; conflict of interest; study design; sample size; source selection; description of the study population (including age, sex, race, ethnicity, and presence and characteristics of survey used); definition and measurement method of the inter- invention; controls; outcomes; results and their variations; and risk-of-bias.

### Data synthesis

The data were organized into evidence tables according to PRISMA guidelines [9]. A descriptive summary was created to determine the study’s characteristics, quality, results, and descriptive statistical analyses to evaluate the outcomes (Table 1).

**Table 1:** Risk of bias assessment

Author(s), year	Compliance	Blinding	Incomplete outcome data	Similarity of groups at baseline	Control of confounding
Moawed et al., 2019 [42]	Self-administered	No	N/M	N/A (Cross-sectional design)	No
Abullais et al., 2020 [31]	Self-administered	No	Of the randomly selected sample of 205 caregivers, 164 completed the study. The response rate was 80%.	N/A (Cross-sectional design)	No. 1- The inter-group statistical comparison for the distribution of categorical variables is done using the Chi-Square test. 2- The inter-group statistical comparison for distribution of means of continuous variables is done using an independent sample t-test for two groups and by an analysis of variance (ANOVA) procedure for more than two groups. 3- The underlying normality assumption was tested before subjecting the study variables to t-test and ANOVA.
Almas et al., 2003 [15]	Self-administered	No	Response rate of 85.5%.	N/A (Cross-sectional design)	No. The data were generated for frequency distributions and Chi-square tests for comparisons.
Kotha et al., 2003 [50]	Self-administered	No	No; To overcome certain rejections, our target was marginally increased to get more than the prescribed sample size.	N/A (Cross-sectional design)	No. 1- An independent sample *t*-test was used to analyze between the parents (mothers and fathers) regarding their knowledge, dietary, and hygiene practices. 2- One-way ANOVA for the other demographic factors was used to analyze the relationship of the parental practices followed by a post hoc analysis to analyze the intragroup influence within mean demographic variables. 3- Chi-square analysis was done to assess how the child was taken to a dentist in relation to demographic variables. Pearson’s correlation was used to correlate parental knowledge and their practices to estimate the interrelationships within themselves.
Al-Abdaly et al., 2019 [49]	?? (Interview and clinical examination)	No	No	N/A (Cross-sectional design)	No. 1- Analysis of variance (ANOVA) was utilized to assess the variations in the mean and standard deviation (± SD) of PLI, GI, PPD, GR and CAL. 2- The Chi-square test was applied to evaluate the relationship between periodontal and oral hygiene status of patients.
Gaffar et al., 2016 [24]	Self-reported	No	197/217 (91%)	N/A (cross-sectional design)	No.
Aldosari et al., 2019 [40]	Self-reported	No	257/469 (55%)	N/A (cross-sectional design)	No. Chi-square test.
Ismaeil et al., 2013 [55]	Self-reported	No	612 (N/M)	N/A (cross-sectional design)	No. Comprehensive descriptive statistics were produced for all demographics and KAP variables.
Srivastava, 2019 [23]	Self-reported	No	228 (N/M)	N/A (cross-sectional design)	No. Post hoc analysis and Chi-square test.
Al-Zahrani et al., 2014 [52]	Self-reported	No	101 (N/M)	N/A (cross-sectional design)	No. Chi-Square test.
Farsi et al., 2020 [22]	Self-reported	No	2586 (N/M)	N/A (cross-sectional design)	No. Paired, unpaired and chi-square test. Tucky’s paired comparison procedures, and correlation coefficients. Wilcoxon-rank sum test and multiple logistic regressions were used to assess the probability of having the disease and risk factor under study.
Al Subait et al., 2016 [21]	Self-reported	No	202/250 (80.8%)	N/A (cross-sectional design)	No. Chi-square test, ANOVA, Bonferroni post-hoc tests and T-test
Al-Shammery et al., 2018 [53]	No	No	813/2200(N/M)	N/A (cross-over design)	No. Nonparametric Mann–Whitney Utest and Wilcoxon’s signedrank test. Shapiro–Wilk test was performed to check the normality distribution
Halawany et al., 2018 [67]	Self-reported	No	1661/1835 (N/M)	N/A (cross-sectional design)	No. One-way ANOVA, Greenhouse-Geisser test, and Wilks’ Lambda. Paired T-test, post-hoc tests. Subtracting the mean difference in the pre- and post-intervention in each class groups.
Mustafa et al., 2018 [16]	Self-reported	No	240/240 (100%)	N/A (cross-sectional design)	No. A simple descriptive analysis was done, and the data were expressed in terms of frequencies and percentages. The collected data were appropriately arranged and analyzed through different computer software applications.
Alshehri et al., 2015 [7]	Self-reported	No	301/425 (93.19%)	N/A (cross-sectional design)	No.
Elsabagh et al., 2018 [48]	Self-reported	No	278/300 (92.6%)	N/A (cross-sectional design)	No. All data was tabulated with frequencies and percentages of answers. Descriptive statistics were performed for the questionnaire items.
Al-Mutairi et al., 2017 [51]	Self-reported	No	108 (54%)	N/A (cross-sectional design)	No.
Ahmad, 2015 [32]	Self-reported	No	114/120 (95%)	N/A (cross-sectional design)	No. Chi-square test.
Al Rasheed et al., 2017 [41]	Self-reported	No	1420/2000 (71%)	N/A (cross-sectional design)	No. Chi-square test.
Ansari et al., 2018 [37]	Self-reported	No	729 (N/M)	N/A (cross-sectional design)	No. Chi-square test.
Alshammary et al., 2019 [29]	Self-reported	No	223/250 (89.2%)	N/A (cross-sectional design)	No. Chi-square test.
Ashour, 2020 [39]	Self-reported	No	247/320 (77%)	N/A (cross-sectional design)	No. Chi-square and Kruskal-Wallis *H* tests.
Al-Shetaiwi et al., 2018 [28]	Self-reported	No	465/500 (N/M)	N/A (cross-sectional design)	No. ANOVA test.
Ansari et al., 2017 [46]	Self-reported	No	794 (N/M)	N/A (cross-sectional design)	No.
Hamasha et al., 2018 [44]	Self-reported	No	519/553 (94%)	N/A (cross-sectional design)	No. Chi square tests.
Mulla et al., 2016 [13]	Self-reported	No	119 (N/M)	N/A (cross-sectional design)	No. Chi-square test.
Al-Johani et al., 2019 [36]	Self-reported	No	200/200 (100%)	N/A (cross-sectional design)	No.
Abu-Hammad et al., 2018 [30]	Self-reported	No	360 (N/M)	N/A (cross-sectional design)	No.
Assery, 2016 [25]	Self-reported	No	252/300 (84%)	N/A (cross-sectional design)	No. Chi square test and *t*-test.
Aljanakh et al., 2016 [33]	Self-administered	No	The response rate in the study was 97%	N/A (cross-sectional design)	No. Chi-square tests were applied to check the association among genders.
Hamasha et al., 2019 [40]	Self-administered	No	Approximately the response rate of 84.5%.	N/A (cross-sectional design)	No. 1- one-way analysis of variance and Bonferroni tests were used to assess differences in the mean number of correct answers among demographic categories.
Sharanesha, 2020 [45]	Self-administered	No	No	N/A (cross-sectional design)	No. Chi-square test.
Aljrais et al., 2018 [38]	Self-administered	No	No	N/A (cross-sectional design)	No. Correlation bivariate test was performed to find the relationship between the DS and PS knowledge, attitude, and practice toward oral health.
Togoo et al., 2012 [2]	Self-administered	No	The response rate of 97%.	N/A (cross-sectional design)	No. Descriptive statistics were obtained and means, standard deviations, and frequency distribution were calculated.
Al-Kheraif et al., 2008 [18]	Clinical examination and Self-administered questionnaire	No	The response rate of the study was 79.2%	N/A (cross-sectional design)	No. Frequency distributions and Chi- square test for statistical evaluation of proportions of the two groups were obtained.
Baseer et al., 2018 [53]	Self-administered	No	No	N/A (cross-sectional design)	No. Kolmogorov–Smirnov and Shapiro– Wilk’s tests
Alshloul, 2021 [12]		No	No	N/A (cross-sectional design)	No. Different differential statistical tests
Wyne et al., 2004 [19]	Self-administered	No	No	N/A (cross-sectional design)	No. Chi-square test
Jaber et al., 2017 [47]	Self-administered	No	No	N/A (cross-sectional design)	No. Chi-square test
Baseer et al., 2012 [14]	Self-administered	No	The response rate of the study was 80.5%	N/A (cross-sectional design)	No. ANOVA, Chi-square tests and z-tests were performed.
Wyne et al., 2015 [34]	Self-administered	No	No	N/A (cross-sectional design)	No. Chi-square test
Wyne, 2007 [27]	Self-administered	No	No	N/A (cross-sectional design)	No. Pearson Chi-Square test and Fisher’s Exact Test
Al-Bader et al., 2006 [26]	Self-administered	No	Response rate of 50%.	N/A (cross-sectional design)	No.
Wyne, 2004 [20]	Self-administered	No		N/A (cross-sectional design)	No. Chi-square test
Awartani, 2009 [54]	Interview	No			No.

KAP, knowledge, attuited and practice; PPD, probing pocket dept; GR, gingival recession; PLI, dental plaque index; GI, gingival index; CAL, clinical attachment loss

### Quality assessment and risk of bias

The methodological quality of the included studies was assessed and recorded in tables according to the PRISMA guidelines, focusing on the following points: 1- Participants compliance: which can vary from self-administrated/self-reported, interviews and clinical examination (2) The blinding factor (3) Incomplete outcome data (4) The similarity between groups at baseline. (5) Assessment of any analysis performed to control for confounding factors that may affect the outcomes (Table 1).

The risk of bias was graded as low, high, or unclear for each domain based on the criteria defined in the Cochrane Handbook for Systematic Reviews of Interventions version 5.1.0. [10].

## Results

### Reviewers’ agreement and kappa score

The k value for inter-reviewer agreement for potentially relevant articles was 0.91 for both abstract and full-text article reviews, indicating an “almost perfect” agreement between the two reviewers [11].

### Study design and populations features

Forty cross-sectional studies and one prospective cohort study were included, as shown in Table 1. The age of participants ranged from 6 to 75 years old. Various populations were studied, including school children students; intermediate and high school students; and university students. Several studies focused on parents of children of various ages, namely: infants (16–40 months), preschool children (2–6 years), and school children (6–12 years). Parents of children with disabilities or disorders such as cerebral palsy were also included. Teachers of primary and secondary schools were also included. Several studies involved outpatients of various medical departments in governmental and private hospitals, including patients with diabetes, those attending dental hospitals, and special-needs patients and caregivers at rehabilitation centers. Multiple studies assessed healthcare providers, including family physicians, pediatricians, nurses, and pharmacists. Five studies included pregnant women to assess their knowledge and practice of oral health.

### Survey tools

Paper- and online-based surveys were the most commonly used tools [2, 7, 12–48].

Other studies used interviews to collect data from their participants [6, 49–51]. Out of 1317 questions asked in these surveys, 553 items were used to measure participants’ knowledge and awareness [2, 6, 7, 12, 14–36, 38, 40–43, 45–52]. Two hundred twenty-six other items were used to determine participants’ attitudes toward oral health [2, 6, 7, 12–14, 17, 19, 21–23, 28, 31, 32, 36, 38, 43, 46–48, 51, 52]. Meanwhile, only 149 items assessed oral hygiene practices [2, 12, 14, 15, 24, 25, 31, 34–36, 38, 42, 47–51].

#### Knowledge outcomes

Studies showed that dental students have significantly higher knowledge score in fluoride beneficial effect of 93.3%, compared to medical students which were of 84.1% and nursing students of 63.6%. (P = 0.027) [21]. Almost one-third of participants (29.4%) knew that plaque is a soft deposit on the teeth, with females having better knowledge (36.5%) compared to males (21.6%) (P = 0.003) [21]. There was a statistically significant mean difference between the four groups in the level of knowledge (F = 4.43, P = 0.005), with the dental students having better knowledge than the other three groups [21]. The majority of students knew the protective effects of fluoride on teeth [21].

Contrastingly, a study found that the mean knowledge scores of dental and pharmacy students were 114.375 ± 26.386 and 48 ± 30.0856, respectively, with a highly significant difference between the two groups (P = 0.000) [38]. A study compared pre-clinical and clinical dental students [46]. Most participants agreed that increased brushing duration would damage the teeth and that brushing alone cannot prevent gum disease [46].

Two studies assessed males’ and females’ knowledge, attitudes, and practices about oral hygiene, which were found to be deficient in many aspects among female college students [48]. Whereas male students demonstrated a good knowledge of basic oral health measures [47]. The majority (63%) knew that poor oral health leads to gum disease, and 59.1% of students were aware of maintaining oral hygiene by using a toothbrush and paste [47].

Among school children, Togoo et al. found that 51.14% of male school children thought that they could keep their gums healthy by daily brushing [2]. Approximately 57.14% of the study populations knew that bleeding gums might indicate gum disease, while 28.24% were unaware thereof [2].

Wyne et al. found no significant difference in oral health knowledge or sources of information concerning age and educational level among male school children [27].

In comparison, a study that designed an interventional program called “oral hygiene awareness” evaluated oral hygiene habits among female Saudi school children; compared to the control group who did not expose to the program, improvements in children’s oral hygiene awareness were observed [18]. The same results were obtained by Baseer et al. who recommended that systematic school-based oral health promotion programs were urgently needed in the Kingdom of Saudi Arabia to target children’s lifestyles and health needs [53]. In addition, another study conducted among school children found that 59.1% of the participants had adequate knowledge [12]. Statistically significant associations were found between age, school type, and students’ educational level and knowledge of oral health care (*P* < 0.05) [12].

Most participants in a study by Farsi et al. knew that toothbrushing helps prevent periodontal disease [22]. Only 33.1% knew that using dental floss helps prevent periodontal disease (P < 0.001) [22]. Additionally, more than half of the participants knew that bleeding on brushing was a primary sign of gingivitis (P < 0.001) [22].

Regarding oral health knowledge during pregnancy, most participants in studies by Moawed et al. and Hammad et al. knew the importance of oral and dental care before pregnancy to reduce dental problems during pregnancy [30, 42] Mowed et al. revealed that gum disease in pregnant women occurs more frequently than in non-pregnant women; however, they did not agree that hormonal changes in pregnancy negatively impact the gum [42]. Gaffar et al. found equivalent results [24].

In addition, oral health knowledge was not significantly associated with reported oral hygiene practices [30]. Moreover, Hammad et al. showed that education level and employment status were significantly associated with a good level of knowledge in oral healthcare of infants (P = 0.000 and 0.002, respectively) [30]. Results of a 22-year comparison survey of dental knowledge at an Al-Jubail antenatal unit showed a decline in dental knowledge and oral health in pregnant women of the current generation, compared with those of the previous generation [25]. Antenatal clinics should educate pregnant women more about the relationship between good oral and fetal health [25].

Regarding oral health awareness in diabetic patients, most participants (81%) were aware that diabetes might increase the risk of oral health problems [54]. Around 75.9% were aware that diabetes might increase the risk for periodontal problems, including gum bleeding and teeth mobility, and 36.3% were aware that diabetes might reduce salivary flow [54]. The primary source of information was the media (31%), followed by dentists and dental hygienists (23%), physicians (21%), and the Internet (16%) [17]. An increase in the level of awareness corresponded with an increase in the knowledge of oral health [55]. However, some studies found the level of awareness and dental health knowledge in diabetic patients deficient [17, 55].

The oral health knowledge score was higher in parents with higher education level. Financial status showed that parental knowledge scores are higher in participants having greater earnings, with a significant correlation to knowledge score [26, 28, 40, 50].

Most parents agreed that good dental health was essential for optimum general health and that regular check-up dental visits help maintain good dental health. Various authors reported that majority of parents agreed with the importance of regular dental visits [7, 19, 20, 28], whereas Alshammary et al. found that only 5.83% of parents answered that the first dental visit should be at 18 months [29]. Wyne et al. concluded that parents’ knowledge of oral health was satisfactory in most areas [26]. The majority (93.3%) of parents could identify “blood on toothbrush during brushing” as a sign of gum disease, with 48.3% attributing it to poor oral hygiene and 45.3% to improper tooth brushing technique [19]. More than half (62.7%) of parents thought cleaning teeth daily keeps gums healthy [19].

Studies conducted among schoolteachers found 75% of male and 72% of female teachers considered irregular tooth brushing a cause of gum disease, with 32% of male and 39% of female teachers not knowing the details regarding the microbial relationship of gum disease [15]. Both groups require more awareness regarding oral health promotion to have a positive role in school oral health education for their students in collaboration with oral health care workers [15].

Results showed that about 80–90% of teachers had sufficient knowledge of the causes and prevention of dental caries and gingivitis [33]. Approximately 94% of teachers agreed that they could play an influential role in oral health promotion, while 96% were found to be interested in performing additional duties as oral health promoters [33].

Another study conducted in Al-Kharj showed that only 38.0% of the schoolteachers responded correctly by saying “plaque means soft debris on teeth,” and only 22.2% said calculus means “hard debris on teeth.” [51] Regarding the squeal of dental plaque, 18.5% of schoolteachers responded that it could cause “staining of teeth” [51]. In contrast, 61.1% of them responded that it might cause “dental caries.” [51].

With regards to brushing, 93.5% felt that it prevented periodontal disease, and 69.4% felt that dental floss prevented periodontal disease [51].

A study conducted in Madinah found a significantly higher (P < 0.001) number of women (80%) had good oral health knowledge compared to men (68%); most of those between the ages of 31 and 40 years showed high scores for oral health knowledge [32]. However, there was no significant relationship between age and knowledge or attitude toward oral health [32].

Statistically significant associations were found between the type of school, age, and years of teaching experience and the knowledge of oral health and its prevention (P < 0.05) [41]. The oral health knowledge of primary school teachers was satisfactory; private primary school teachers had better knowledge than government school teachers. It is recommended that the effectiveness of oral health education programs in primary schools be evaluated [41].

Oral health knowledge among primary school teachers is suitable for school-based oral health programs [43]. Administrative barriers were the most significant barriers to implementing a school oral health program [43]. There is a need for concerned school authorities and health policymakers to address these barriers and promote oral health in the community [43].

Al-Johani and Elanbya [36] found that only 15% of teachers regularly discuss oral health topics with their students [36]. Of those respondents, 74.5% think treating tooth caries in primary teeth is necessary, and 70.5% think dental health education should be included in the primary school curriculum [36].

Caregivers were also included in several studies. The majority of them chose the correct answers to the questions that evaluated their periodontal and oral health knowledge and awareness, except the question regarding when to change the toothbrush; 51% and 59% of caregivers in group I (Visual impairment group) and III, respectively, chose the wrong answers compared with 55% of them who chose the correct answers in group II (moderate mental retardation group) [49]. Generally, caregivers had good periodontal and oral health knowledge and awareness, particularly in group II [49].

A great majority were aware of the importance of healthy teeth in relation to chewing (90%), esthetics (80%), and speech (68.3%) [34]. Similarly, almost all (95%) caregivers were aware of the importance of good dental health for optimal general health [34]. Approximately three in every four (73.3%) workers knew that one should visit a dentist twice a year for regular check-ups [34]. It can be concluded that the special health care workers in the disabled children’s center generally had satisfactory oral health knowledge and practices [34].

The level of knowledge was significantly higher among the younger caregivers compared to the older age group (P < 0.05) [31]. Caregivers in the 20–29-year age group demonstrated better knowledge than other age groups (P < 0.05) [31]. The level of knowledge based on gender and experience did not differ significantly among groups (P > 0.05) [31]. The group of caregivers exhibited a significantly good level of knowledge with an education level above that of a high school level compared to caregivers with a level of education below a high school level (P < 0.05) [31].

Most participants said they had not visited a qualified dentist in the past, and many did not know the correct way to brush their teeth [16]. Hence, deaf and hard-of-hearing individuals are lacking [16].

Among healthcare professionals, doctors showed a higher mean knowledge score than other health professionals, which yielded statistically significant differences (*P* < 0.05) [14].

#### Practice outcomes

Regarding oral hygiene practices among university students, a study by Aljrais et al.reported that 113 (75.3%) and 127 (84.6%) of dental and pharmacy students at Riyadh Elm University, respectively, brushed their teeth 2–3 times a day [38]. The comparison between pharmacy and dental students was statistically insignificant (P = 0.07) [38].

On the other hand, Jaber et al. found that male Qassim University students showed poor oral practices; almost 71.3% brushed their teeth once daily [47].

Regarding oral hygiene practice among school children, three studies showed that more than half of the population brushed their teeth using toothbrushes and toothpaste [2, 12, 22]. Farsi et al. found that tooth brushing among intermediate and high school students living in Jeddah was the most frequent method used (83.8%) [22]. In a study by Alshloul, the most common hygiene aid used among school children in Abha was using a toothbrush with toothpaste (78.3%) [12]. Additionally, Togoo et al. found that 58.4% of the participants brushed their teeth using a toothbrush and toothpaste [2].

In two studies, toothpaste was considered a separate measurement. According to Srivastava, only 4.9% of rural and urban school children in the Al Qassim region used toothpaste to maintain oral hygiene (P = 0.009) [23]. On the other hand, Farsi et al. found that toothpaste is the primary material used for cleaning teeth (91.1%) [22].

Two studies found that 32.1% and 39.9% of the participants used *miswak* (a natural aid to replace toothbrush) as their teeth-cleaning aid [2, 22]. On the other hand, only one study showed that 85.1% of the rural and urban school children in the Al Qassim region used *miswak* (P = 0.009) [23]. Two studies showed that only a minority used dental floss. According to Farsi et al. dental floss it is the least-used method (19.6%) [22]. Similarly, in another study, only 2.3% of the participants used dental floss [2].

Four studies that assessed the oral health practices of pregnant women found varying teeth brushing frequencies; 83.5% cleaned their teeth daily and considered brushing and using toothpaste essential for pregnant women [42]. Gaffar et al. found that 51.5% brushed at least twice daily [24]. Moreover, in a study by Assery et al., 33.3% of respondents reported brushing their teeth twice daily [25]. Assery et al. performed a 22-year comparison survey with 1996 data, showing a nearly 50% increase in the percentage of pregnant women who brushed their teeth once or less per day (from 23 to 7.6%; proportional t-test, P < 0.05) [25]. On the other hand, there was a significant decrease in the percentage of women who brushed their teeth more than once a day (from 77 to 52.3%; proportional t-test, P < 0.05) [24]. Regarding the use of other dental aids like dental floss and *miswak*, Gaffar et al. mentioned in their study that 43.8% sometimes flossed and 47.7% sometimes used *miswak* [24].

Oral hygiene practices in patients with diabetes varied in different studies. The study stated that 83.2% brushed their teeth [56]. In a study by Awartani, 80% did not brush [54]. Moreover, Basil and Rakan found that 45.6% regularly brushed their teeth once daily [17]. Different age and gender groups did not show any statistically significant difference in brushing habits and in the level of awareness of the increased risk of oral health problems for patients with diabetes (P > 0.05) [17]. The use of other dental aids by patients with diabetes was mentioned only in the Basil and Rakan’s study, where 10.4% flossed their teeth regularly, and 11.5% used mouth rinse at least once a day [17].

Alshammary et al. assessed the oral health practices of parents of children and found that approximately 71.75% of participants used a toothbrush with a fluoride-containing paste [29]. Al-Shetaiwi et al. found that 40% of the participants answered that their child did not brush their teeth [28]. moreover, 31% regularly guided their primary school children in tooth brushing, and 12% confirmed that their children also regularly cleaned their tongues [45]. Wyne et al. found that almost all 98.7% parents responded that they could maintain excellent dental health in their children by supervising their tooth brushing, reducing sugary food intake, and visiting the dental clinic regularly [19]. Furthermore, Sharanesha and Bhari found that 86% of parents included green leafy vegetables regularly in their child’s diet [45]. In addition, Kotha et al. found that parents with good knowledge follow better dietary practices with their children, which was statistically significant (P < 0.05), particularly with respect to hygiene practices (P < 0.001) [50]. These findings are supported by Ansari et al., who showed that mothers with advanced education have significantly superior knowledge regarding oral hygiene practices and deciduous teeth [46].

Studies conducted among schoolteachers to assess oral practices found that most teachers used toothpaste and toothbrushes. Al-Johani and Elanbya found that 45.4% of the participants used *miswak* to clean their teeth [36]. Regarding the timing and frequency of teeth brushing, Almas et al. found that tooth brushing three times a day was more common among female teachers (33.5%) than male teachers (18.6%) (P = 0.000) [15]. On the other hand, Al-Johani and Elanbya found that 44.5% of teachers brushed their teeth twice daily [36].

Healthcare professionals practice oral health; two studies showed that the majority cleaned their teeth with a toothbrush and toothpaste [14, 34] Less than 10% used *miswak* and toothpicks for oral hygiene [14, 34]. Females were significantly more likely to use dental floss than male health professionals (P < 0.05) [14, 34]. Similarly, university graduates were significantly more likely to use dental floss and mouthwash than diploma holders (P < 0.05) [14, 34].

Regarding other dental aids, Baseer et al. found that less than 50% of health professionals used mouthwash and dental floss [14].

## Discussion

Poor oral health can cause numerous infectious and degenerative diseases that may adversely influence general health and increase healthcare costs. The need for an effective method to improve oral health is obligatory [56]. Several factors showed to affect the overall knowledge and awareness of oral health: the type of school, age, and years of teaching experience with knowledge of oral health and its prevention were significantly influential [41]. Oral health awareness was assessed among primary school teachers in both governmental and private sectors [41].

Total participants from all the included studies such as school children, [2, 12, 22] school teachers [36, 51] and healthcare professionals [14, 34] reported brushing their teeth with toothbrush and toothpaste. With regards to the frequency of teeth brushing many populations included in this study showed positive compliance. For instance, female teachers brushed three times a day [15]. Almost half of the university students [13, 21, 48] and caregivers [31, 49] brushed their teeth twice a day. In contrast, patients with diabetes and pregnant women brushed their teeth with varying frequencies. Majority of the included populations showed fewer compliance in regards to other dental aids. For example, a minority of school children [2, 22], diabetic patients [17] and healthcare professionals [14] used dental floss. Almost half of pregnant women used floss and *miswak* only sometimes [24]. In contrast, university graduates were significantly more likely to use dental floss and mouthwash than diploma holders. Moreover, females were significantly more likely to use dental floss compared with male health professionals. As part of the Saudi culture, the use of *miswak* is more common among rural and urban school children in the Al Qassim region [23]. Less than half of school teachers used only *miswak* for cleaning their teeth [51].

Many different approaches exist to prevent dental diseases, of which health education is the most cost-effective method [57]. The main focus in improving the knowledge and practice of oral hygiene is through oral health education and promotion interventions (OHEPIs), which improve oral health behaviors that can enhance oral and clinical health [58].

Dental health education can be conveyed to individuals and groups in various settings; for example, dental practices, the workplace, schools, day-care centers, and residential settings for older adults [59]. A strategy implemented by the World Health Organization (WHO) for the prevention and promotion of oral health has operated upon the basis of increasing the awareness of oral health worldwide as an essential factor of public health and quality of life in its Global Oral Health Program [57, 59].

It has been recognized that using mobile technologies, known as mobile health (mHealth), is effective for improving health [60]. The use of mHealth combined with conventional oral health education programs has been postulated to increase compliance amongst adolescent patients compared with verbal instructions of oral hygiene alone [61]. A novel and effective way of delivering health information to a large population is through educational apps due to their widespread use and powerful technological advances [61]. Studies by Zahid et al. and Scheerman et al. both used mobile apps to promote oral hygiene [61, 62]. Zahid et al. stated that participants were instructed to use the app twice daily for three months [61]. For the conventional education group, a 20-minute lecture session on good oral hygiene practices was delivered by a dental hygienist using a whiteboard, markers, presentation slides, and dental teeth models [62].

Another method that aided in improving oral hygiene was mentioned in a study by Zotti et al. they evaluated the influence of an app-based method in a protocol for oral hygiene maintenance in a group of adolescent patients wearing fixed multibracket appliances [63]. Standardized oral hygiene instructions were delivered to participants via WhatsApp (WhatsApp Inc. Facebook, Inc. 2020. Available from: https://whatsapp.com). Incorporating new social technologies in a standard oral hygiene motivation protocol effectively improves oral health status during orthodontic multibracket treatment [63].

Another study was done by Bowen et al., which aimed to evaluate the associated effect of text message reminders sent directly to patients on their oral hygiene compliance using planimetry as a tool to measure plaque [64]. The results demonstrated that sending encouraging text messages reminding participants of good oral hygiene resulted in a detectable reduction in plaque surface area over time [64].

Many health-promoting interventions that successfully changed health behavior included methods that targeted different phases of the behavior change process, that is, the process of behavioral initiation and maintenance, similar to providing health‐risk information and self‐monitoring of behavior [61].

Another study by Dusseldorp et al. focused on enlarging the effectiveness of health-promoting interventions by merging multiple behavior change techniques (BCTs), such as prompt intention formation and providing feedback on performance, which can be reflected as the atomic measures of intervention [65]. For instance, a study conducted among cooperative children with autism in Jazan, Saudi Arabia, used applied behavioral analysis techniques using 15 videos in Avatar technology [66]. The results showed significant improvements in the behavior and knowledge of cooperative autistic children after applying the interventions above [66].

A study by Halawany et al. aimed to assess the effectiveness of an oral health education intervention among female primary school children in Riyadh, Saudi Arabia [67]. After distributing a pre-intervention questionnaire, the program consisted of three elements: a four-minute animation video, a lecture presentation, and four educational booths [67]. This study showed a significant improvement in oral health knowledge and self-reported behavior. A different study investigated the effectiveness of mobile applications compared to conventional educational lectures on high school students’ oral hygiene knowledge and behavior in Jeddah city, Saudi Arabia [62]. According to their findings, mobile applications (e.g.: Brush DJ) and conventional lectures were equally effective in improving oral health knowledge, attitude, and behavior [62]. However, participants who used the Brush DJ app showed better frequency and duration in teeth brushing [62]. Another mobile application used a phone-based education program to assess its effect on oral health knowledge among mothers in Riyadh and Najran cities, Saudi Arabia; significant improvement was noted among mothers in both regions [68]. Moreover, the application was more effective in mothers with more than one child than in first-time mothers [68]. However, a systematic review was conducted to assess the effectiveness of oral health education using the mHealth approach of parents for improving their children’s oral health, revealing a low–very low certainty of evidence proving that the mHealth approach could improve parents’ oral health knowledge [69]. Therefore, further studies on this matter with regard to designing better educational content are warranted [69].

Another systematic review aimed to evaluate the effectiveness of mobile applications and text messages, compared with conventional oral hygiene instructions, for improving oral health knowledge or reducing gingival inflammation when delivered to young children, adolescents, adults, and mothers [70]. Better results were obtained using mobile technology in 13 out of 15 studies [70]. Also, a significant improvement in dental plaque control and gingival bleeding was reported for groups that received the mHealth strategy [70].

## Conclusions

Effective oral health education and oral hygiene practices are the keys to reaching one of the ultimate goals of improving oral health among the population in the country. The present systematic review revealed a strong association between oral health knowledge and practices since the higher the knowledge level, the better the practice. Therefore, new technologies and strategies must be tested to have an effective oral health system.

## Data Availability

The datasets used and/or analyzed during the current study are available from the corresponding author on reasonable request.

## List of abbreviations

BCTs: behavior change techniques
DS: dental students
mHealth: mobile health
MS: medical students
NS: nursing students
OHEPIs: oral health education and promotion interventions
PRISMA: Preferred Reporting Items for Systematic Reviews
WHO: World Health Organization
